# Discovery of actinomycin derivatives with improved selectivity against malaria parasites from a *Streptomyces* culture library

**DOI:** 10.1038/s41429-026-00924-0

**Published:** 2026-05-07

**Authors:** Aiko Teshima, Awet Alem Teklemichael, Asahi Hirata, Momoko Akimoto, Mayumi Taniguchi, Rukman Muslimin, Sho Ogaki, Alimuddin Ali, Toshihiro Suzuki, Shusaku Mizukami, Kenji Arakawa

**Affiliations:** 1 Program of Biotechnology, Graduate School of Integrated Sciences for Life, Hiroshima University, 1-3-1 Kagamiyama, Higashi-Hiroshima, Hiroshima 739-8530 Japan; 2 Hiroshima Research Center for Healthy Aging (HiHA), Hiroshima University, 1-3-1 Kagamiyama, Higashi-Hiroshima, Hiroshima 739-8530 Japan; 3 Department of Immune Regulation, Global Infectious Diseases Division, Institute of Tropical Medicine, Nagasaki University, 1-12-4 Sakamoto, Nagasaki, Nagasaki 852-8523 Japan; 4 School of Tropical Medicine and Global Health, Nagasaki University, 1-12-4 Sakamoto, Nagasaki, Nagasaki 852-8523 Japan; 5 Department of Biology, Universitas Negeri Makassar, Jl. Dg Tata Raya Parangtambung, Makassar, South Sulawesi 90223 Indonesia; 6 Department of Fermentation Sciences, Faculty of Applied Biosciences, Tokyo University of Agriculture, 1-1-1 Sakuragaoka, Setagaya, Tokyo 156-8502 Japan

**Keywords:** Natural product synthesis, Drug discovery and development

## Abstract

Our group explores the antimalarial compounds from the *Streptomyces* culture library. Pilot screening showed significant antimalarial activity in *Streptomyces antibioticus* HUT6035 (synonym of strain NBRC3117). We here isolated antimalarial compounds through large-scale fermentation and subsequent purification using chromatographies. Strain HUT6035 accumulated actinomycins X_2_ and X_0ß_, which were 4-oxo and 4-hydroxy proline derivatives of actinomycin D, respectively. The former showed an antimalaria activity (IC_50_ = 0.241 nM) and a cytotoxicity (CC_50_ = 6.71 nM), while the latter showed IC_50_ = 7.69 nM and CC_50_ = 818 nM. Compared with commercially available actinomycin D (IC_50_ = 0.469 nM and CC_50_ = 6.71 nM), both actinomycins X_2_ and X_0ß_ have significant selectivity index (SI) values (CC_50_/IC_50_) with 27.9 and 106. These SI values were two- and seven-fold preferential against that of actinomycin D (SI = 14.3). Thus, actinomycin derivatives are potential antimalarial agents.

## Introduction

The filamentous Gram-positive bacterial genus, *Streptomyces*, is well characterized as the prolific producer of secondary metabolites with a vast array of significant biological activities [1]. Bioactive molecules controlling physiological functions in various organisms have been discovered through extensive screening of the *Streptomyces* culture library [2–8]. Our group also discovered an 18-membered macrolide borrelidin with a necrotic activity against potato tubers [9] and a bipyrrole compound 4-methoxy-2,2′-bipyrrole-5-carbaldehyde as a strobilation inhibitor against moon jellyfish without inducing cytotoxicity [10].

Malaria is a major global infectious diseases caused by the mosquito-borne *Plasmodium*. Among the five species that infect humans, *Plasmodium falciparum* is responsible for most severe and fatal cases. In 2024, there were almost 282 million estimated malaria cases across 80 malaria-endemic countries. Furthermore, 610,000 deaths were associated with malaria in 2024. Unexpectedly, there is an increase of 12 million compared to 2023 [11]. Sustainable discovery and development of new antimalarial drugs are key element in combating malaria. However, the overuse or insufficient use of antimalarial drugs sometimes lead to the emergence of drug-resistant strains. To reduce the occurrence of malaria disease, structural modification of parent compounds and extensive exploration of bioresources are considered to be practical strategies. Chloroquine, a first-generation antimalarial agent, is associated with considerable side-effect and its overuse led to emerge drug-resistant strains. To improve its safety profile, hydroxychloroquine was developed by introducing a hydroxyl group, while largely retaining the antimalarial activity of chloroquine. However, hydroxychloroquine does not overcome chloroquine resistance. To address drug-resistant malaria, structurally distinct compounds such as mefloquine were subsequently developed. Thus, both the structural modification of parent compounds and the development of new chemical scaffolds are essential to improving biological activity and overcoming drug resistance in malaria treatment. Regarding extensive exploration of bioresources, we have previously discovered antimalarial compounds in traditional Kampo medicine [12] and in the leaves of *Morinda morindoides* [13, 14]. *Streptomyces* strains are potential antimalarial agents. For example, a phosphonate FR900098 was isolated from *Streptomyces rubellomurius* [8, 15] and an α-pyridone compound iromycin from *Streptomyces* sp. RBL-0292 [16]. Furthermore, some of our *Streptomyces* culture libraries showed promising antimalarial activity against both chloroquine/mefloquine-sensitive (3D7) and -resistant (Dd2) cell lines of *P. falciparum* [17]. Among the screened libraries, *Streptomyces antibioticus* HUT6035 (a synonym of strain NBRC3117) showed the highest activity and lowest cytotoxicity.

In this study, we isolated antimalarial compounds through the large-scale fermentation of the strain HUT6035 and subsequent purification using various chromatography techniques. Strain HUT6035 accumulated actinomycins X_2_ and X_0ß_, which are the 4-oxo and 4-hydroxy proline derivatives of actinomycin D, respectively. Their antimalarial activity, cytotoxicity, and selectivity index (SI) were investigated, and the results are described herein.

## Materials and methods

### Bacterial strain used in this study and preparation of the culture extract

*Streptomyces antibioticus* strain HUT6035 was cultured in YM medium (0.4% yeast extract, 1.0% malt extract, and 0.4% D-glucose, pH 7.3) at 28 °C with 120 rpm (revolutions per minute) for 5 days, according to our standard protocol [18].

### Spectroscopic instruments

The active components of strain HUT6035 against *P. falciparum* strains 3D7 and Dd2 were analyzed using electrospray ionization-mass spectrometry (ESI-MS) and nuclear magnetic resonance (NMR). The ESI-MS spectra were obtained using an Orbitrap Eclipse^TM^ Tribrid^TM^ mass spectrometer (Thermo Fisher Scientific, Waltham, MA, USA). High-resolution ESI-MS was performed in positive ion mode. The NMR spectra (600 MHz for ^1^H NMR and 150 MHz for ^13^C NMR) were recorded using an ECZL600G spectrometer (JEOL, Tokyo, Japan) equipped with a field gradient accessory. The NMR chemical shifts were recorded as *δ* values in ppm. The coupling constants in ^1^H NMR were shown as *J* values in Hz. Deuteriochloroform was used as the solvent. Chemical shifts were recorded in *δ* value based on solvent signals (*δ*_C_ = 77.0 in CDCl_3_) or the internal standard tetramethylsilane (*δ*_H_ = 0).

### Isolation of actinomycins from *Streptomyces antibioticus* strain HUT6035

The culture supernatant of HUT6035 cells (6 L) was extracted twice using an equal volume of ethyl acetate (EtOAc). The combined organic phases were dried (Na_2_SO_4_), filtered, and concentrated *in vacuo*. The crude extract was purified by gel filtration chromatography on Sephadex LH-20 (GE Healthcare, Chicago, IL, USA) in MeOH. All fractions (1 mL each; total 50 fractions) eluted with MeOH were subjected to a bioassay using *P. falciparum* 3D7 and Dd2 for antimalarial activity and primary Adult Mouse Brain (AMB) cells for cytotoxicity according to our previous report [17]. The fractions containing the active component(s) with antimalarial activity were combined, and the resulting reddish residue was further purified by silica gel chromatography in CHCl_3_–MeOH = 100:1–50:1 (v/v) to obtain two active components. These two components, HUT6035A and HUT6035B, appeared as reddish spots on TLC at Rf = 0.50 and 0.40 at CHCl_3_–MeOH = 15:1 (v/v), respectively. Compounds HUT6035A and HUT6035B were identified as actinomycin X_2_ and actinomycin X_0ß_, respectively, according to the reported data [19–22].

HUT6035A ( = actinomycin X_2_): The ^1^H and ^13^C NMR assignments are shown in Table S1. HRMS (positive ESI): *m*/*z* calculated for C_62_H_84_N_12_O_17_Na: 1291.5970 [M+Na]^+^; observed: 1291.5984.

HUT6035B ( = actinomycin X_0ß_): The ^1^H and ^13^C NMR assignments are shown in Table S2. HRMS (positive ESI): *m*/*z* calculated for C_62_H_86_N_12_O_17_Na: 1293.6126 [M+Na]^+^; observed: 1293.6140.

### Bioassay for the antimalarial activity and the cytotoxicity

Malarial parasite cultures and AMB cells were previously described [17]. The detailed experimental procedure is described in the Supplementary data. Antimalarial activity and cytotoxicity were evaluated at 50% inhibitory concentration (IC_50_) and 50% cytotoxic concentration (CC_50_), respectively. We here analyzed these activities on purified actinomycin X_2_ (**1**), actinomycin X_0ß_ (**2**), and commercially available actinomycin D (**3**). The SI is calculated as CC_50_/IC_50_.

### Time-course production of actinomycins in strain HUT6035

The EtOAc extract from a 100-mL culture of strain HUT6035 (1-5 days of cultivation) was dissolved in MeOH (1 mL) and analyzed by HPLC and ESI-MS. For HPLC analysis, each aliquot (10 µL) was applied on a COSMOSIL CHOLESTER column (4.6 I.D. x 250 mm, Nacalai Tesque, Kyoto, Japan) and eluted with 70% aqueous MeOH at a flow rate of 1.0 mL min^–1^. The eluate was monitored at 450 nm using an MD-2010 multiwavelength photodiode array detector (JASCO Corporation, Tokyo, Japan). Owing to the overlapping peaks for **1** and **3** in the HPLC chromatogram, the production yields of **1** and **3** were evaluated by ESI-MS through the peak intensities of the monoisotopic signals. Purified natural **1** and **2**, and the commercially available **3**, were analyzed in the same manner.

### DNA sequencing and assembly

*Streptomyces* total DNA was prepared according to a previously modified protocol [23]. Draft genome sequencing was performed using a paired-end sequencing strategy (2 × 300 bp) on an Illumina NextSeq 1000 platform (Illumina, Inc., San Diego, CA, USA). De novo assembly of the raw genome sequencing data was performed using SPAdes 4.2.0 [24]. The actinomycin biosynthetic gene cluster (BGC) was identified using antiSMASH ver. 8.0.3 [25], and its annotated sequence was deposited in the DDBJ/ENA/GenBank database under the accession number: LC912581.

Detailed experimental procedure for sample preparation of *Streptomyces* total DNA and next-generation sequencing is described in the Supplementary data.

## Results and discussion

### Isolation of actinomycins from *Streptomyces antibioticus* strain HUT6035

In our pilot screening of 28 actinomycete culture extracts, 17 samples showed parasite growth inhibition of more than 50% at a concentration of 50 µg/mL [17]. Among them, the culture extract of *Streptomyces antibioticus* strain HUT6035 showed the highest antimalarial activity with an IC_50_ value of 0.09 and 0.22 µg/mL against 3D7 and Dd2, and SI of 188 and 73.7, respectively [17]. Hence, we isolated antimalarial compound(s) with high SI values from strain HUT6035.

The culture extract (6-L culture) of HUT6035 was purified using Sephadex LH20 and silica gel chromatography, with a bioassay using *P. falciparum* strains 3D7 and Dd2 for antimalarial activity and AMB cells for cytotoxicity. Two reddish spots detected at Rf = 0.50 and 0.40 in CHCl_3_–MeOH = 15:1 (v/v) showed significant antimalarial activity. They were designated as HUT6035A and HUT6035B (average isolation yields of 25 mg and 3.3 mg per L, respectively), and further analyzed by ESI-MS and NMR analyses [19–22].

**Fig. 1:**
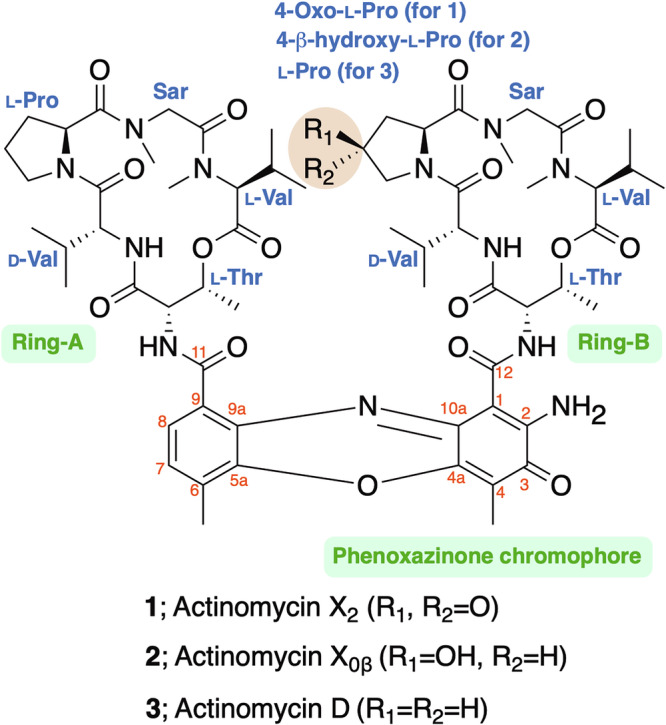
Structures of actinomycin X_2_ (**1**) and actinomycin X_0ß_ (**2**) isolated from *Streptomyces antibioticus* HUT6035. The representative actinomycin derivatives, actinomycin D, was also described as compound **3**. Sar, sarcosine

In high-resolution ESI-MS analysis, HUT6035A was established as C_62_H_84_N_12_O_17_. The ^1^H and ^13^C NMR assignments of HUT6035A are summarized in Table S1 (Supplementary Materials). In the ^13^C NMR spectrum of HUT6035A, 62 signals were detected and classified as 16 methyls, 7 methylenes, 16 methines, and 23 nonprotonated carbons. Their connectivity was further confirmed using 1D and 2D NMR techniques including HMQC and HMBC spectra (Figs. S1–S2), in good agreement with actinomycin X_2_ ( = actinomycin V) (Fig. 1; compound **1**) [19–22]. In contrast, the molecular formula of HUT6035B was determined to be C_62_H_86_N_12_O_17_, with two protons larger than that of HUT6035A. The NMR assignment (Table S2) and spectra of HUT6035B (Figs. S3–S4) showed good agreement with the reported data for actinomycin X_0ß_ (Fig. 1; compound **2**) [21, 22].

### Significant selectivity index of actinomycin derivatives for antimalarial activity

The two actinomycin derivatives together with commercially available actinomycin D (Fig. 1) were subjected to an antimalarial activity assay using *P. falciparum* strain 3D7. To determine the selectivity index (SI) of the actinomycins, their cytotoxicity was investigated using AMB cells (Table 1). Actinomycin X_2_ (**1**) showed an antimalarial activity (IC_50_ = 0.241 nM) and a cytotoxicity (CC_50_ = 6.71 nM), while actinomycin X_0ß_ (**2**) showed IC_50_ = 7.69 nM and CC_50_ = 818 nM. Compared with commercially available actinomycin D (**3**) (IC_50_ = 0.469 nM and CC_50_ = 6.71 nM), both **1** and **2** had significant selectivity index (SI) values (CC_50_/IC_50_) of 27.9 and 106, respectively. The SI value is important for the safety of human cells, and the evaluation of drug candidates. These SI values were 2- and 7-fold higher than those of actinomycin D, respectively (SI = 14.3). Therefore, actinomycin derivatives are potential antimalarial agents. Alenazi et al. reported the antimalarial activity of actinomycin D, however, they did not mention the SI value [26]. In terms of the SI value, actinomycin D is considered to be a less promising candidate for an antimalarial drug. This tendency corresponds to our preliminary screening of Indonesian *Streptomyces* sp. AA018, an actinomycin D overproducer, was isolated from Karst in Sulawesi Island as part of our international collaborative project [27, 28]. The culture extract of strain AA018 was removed from the list of potential antimalarial drug candidates due to its low SI value. These findings indicate that the structural modification of the mother compound may lead to increased SI values, which are important for clinical antimalarial agents. Similarly, our group reported that structural modification of lankacidin through chemical modification led to improved antitumor activity by filling the binding pocket in microtubule [29, 30]. Hence, structural variants are worth studying for their biological activity.

**Table 1:** In vitro antimalarial activity and cytotoxicity of actinomycin derivatives^a^

Compounds	IC_50_ (nM)^b^	CC_50_ (nM)^b^	SI^b^
actinomycin X_2_ (**1**)	0.241	6.71	27.9
actinomycin X_0ß_ (**2**)	7.79	818	106
actinomycin D (**3**)	0.469	6.71	14.3

^a^The in vitro antimalarial activity of actinomycetes secondary metabolites extracts against strain 3D7 of *P. falciparum*, and cytotoxicity against Adult Mouse Brain Cells (AMB)

^b^IC_50_ 50% inhibitory concentration, CC_50_ 50% cytotoxic concentration, SI selectivity index (CC_50_/IC_50_). The value of IC_50_, CC_50_, and SI is taken from two independent experiments performed in duplicate wells

### Time-course production of actinomycins in strain HUT6035

**Fig. 2:**
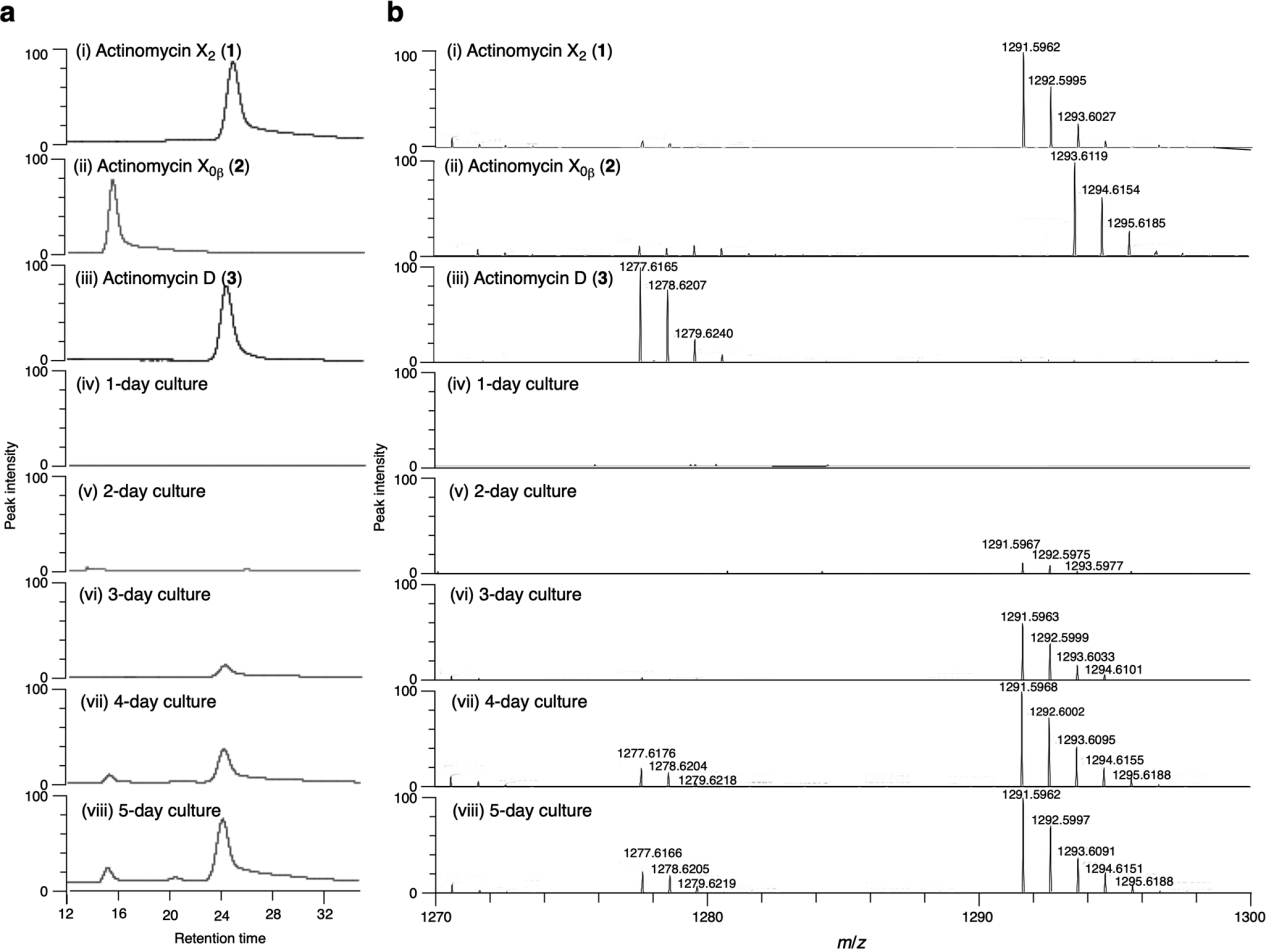
Time-course production of actinomycins in strain HUT6035. Strain HUT6035 was cultivated and analyzed the production of actinomycins at 1–5 days periods using HPLC **a** and ESI-MS **b** analyses. (i) actinomycin X_2_ (**1**), (ii) actinomycin X_0ß_ (**2**), (iii) actinomycin D (**3**), (iv) 1-day culture, (v) 2-day culture, (vi) 3-day culture, (vii) 4-day culture, and (viii) 5-day culture

**Fig. 3:**
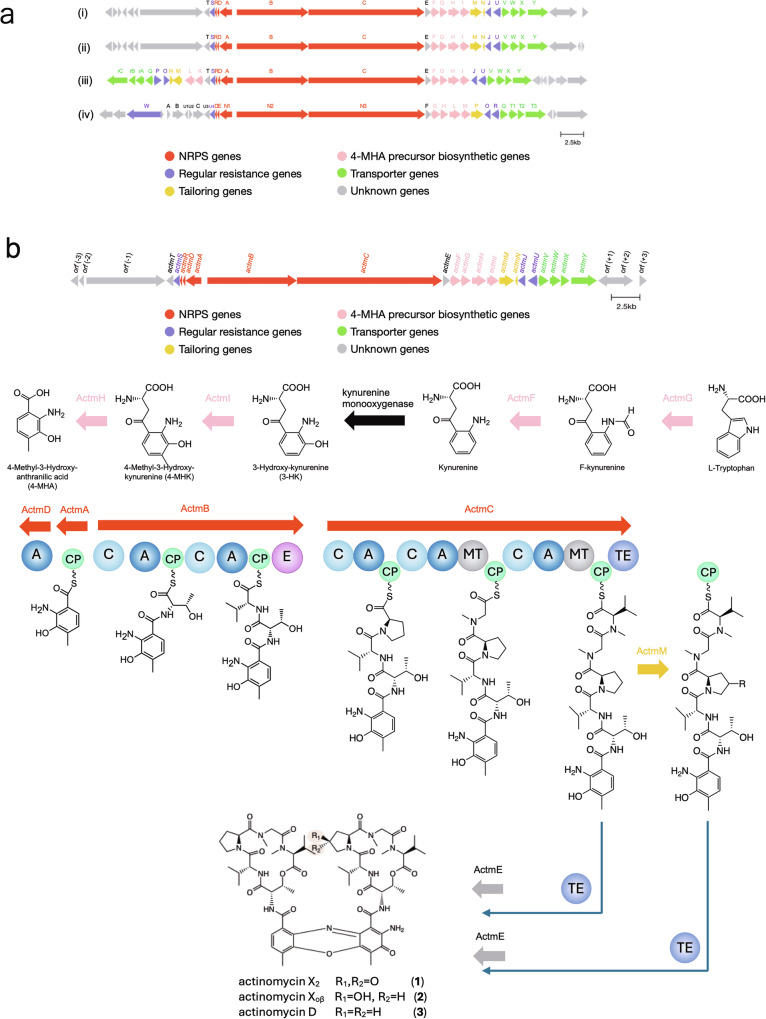
Biosynthetic gene cluster for actinomycins in strain HUT6035. **a** Gene organization of actinomycin biosynthetic gene (*actm*) cluster in strain HUT6035 (panel i) and other producing strains, *Streptomyces antibioticus* IMRU3720 (panel ii), *Streptomyces chrysomallus* ATCC11523 (panel iii), and *Streptomyces costaricanus* SCSIO ZS0073 (panel iv). **b** Possible biosynthetic pathway of actinomycins

Culture extracts of strain HUT6035 (1–5 days of cultivation) were analyzed using HPLC and ESI-MS. Equal volumes of crude extracts (10 µL of 1 mL MeOH solution) at every period were injected for normalization, and their productivity was estimated by the peak area between peak curves and baseline. In our HPLC condition (Fig. 2a), actinomycins X_0β_ (**2**) elutes at 15.5 min, and actinomycin X_2_ (**1**) and actinomycin D (**3**) elute at 24.5 min with overlapping. Hence, the production ratios of **1** and **3** were evaluated based on the peak intensities in their ESI-MS spectra monoisotopic signals at *m*/*z* 1291.60 for **1,** *m*/*z* 1277.62 for **2**, and *m*/*z* 1293.61 for **3**. Compound **1** accumulated prior to **2** and **3** in strain HUT6035 (Fig. 2). The production ratios of **1**–**3** were 100:0:0 (2-days culture), 100:5:5 (3-days), 100:15:20 (4-days), and 100:20:20 (5-days), respectively, based on the peak intensities of their monoisotopic signals. Under our culture conditions for strain HUT6035, actinomycin derivatives rather than **1**–**3** could not be detected by either HPLC or ESI-MS analyses (Fig. 2). Compounds **1**–**3** were designated actinomycin X (a mixture of actinomycins) in *S. antibioticus* IMRU3720 [31]. Genome sequencing of strain HUT6035 using an Illumina NextSeq 1000 sequencer revealed the presence of an actinomycin BGC. Reported actinomycin BGCs are shown in Fig. 3a. The actinomycin BGC (*actm* cluster) in HUT6035 has an end-to-end similarity to that of *Streptomyces antibioticus* IMRU3720 (Fig. 3a, panel ii) [31]. They have a variety of BGC compared to *Streptomyces chrysomallus* ATCC11523 (Fig. 3a, panel iii) [31] and *Streptomyces costaricanus* SCSIO ZS0073 (Fig. 3a, panel iv) [32]. The substrate specificity of A-domain was predicted using a web-based NRPS A-domain predictors [PARAS (Predictive Algorithm for Resolving Adenylation domain Selectivity): https://paras.bioinformatics.nl/data_annotation] [33]. Substrate specificity determining residues in two NRPS enzymes ActmB and ActmC of strain HUT6035 were similar to those in *S. antibiotics* IMRU3720, rather than those in S. *chrysomallus* ATCC11523 (Fig. 3b). ActmM functions as a cytochrome P450 enzyme in coordination with ferredoxin ActmN, indicating the oxidation of ring-B in the biosynthesis of **1** and **2**. ActmF presumably catalyzes the condensation of two pentapeptide chains into the phenoxazinone core during actinomycin biosynthesis (Fig. 3b) [32, 34, 35]. The following features are unique to actinomycin biosynthesis: (1) regiospecific oxidation of a proline residue occurs only in ring-B, not in ring-A, and (2) oxidized ring-B is preferentially coupled with ring-A through condensation to a phenoxazinone core in strain HUT6035. This observation was also reported by Wang et al. [22]. The preferential production of **1** and **2** over **3** indicates the rich building blocks of the oxidized pentapeptide chains, rather than the pentapeptide chain with non-oxidized proline, which is used for ring-A in **1**–**3** and ring-B in **3**. The variable production of actinomycins with an oxidative degree strongly suggests relaxed substrate specificity for the condensation reaction between the two peptide chains and the phenoxazinone core.

## Conclusion

We have revealed that actinomycin derivatives, actinomycins X_2_ and X_0ß_, produced by strain HUT6035, showed higher SI values (27.9 and 106) compared with their mother compound, actinomycin D (14.3). Value-added derivatives are available through natural variants or artificial conversion (e.g., chemical synthesis and enzymatic modifications). Thus, structural variants are important resources for improving biological activity.

## Supplementary information

SUPPLEMENTAL MATERIAL
