# Supplementary material for: Discovery of actinomycin derivatives with improved selectivity against malaria parasites from a Streptomyces culture library
Source: J Antibiot (Tokyo). 2026 May 7;79(7):461–8. doi: 10.1038/s41429-026-00924-0 (PMC13310753; doi:10.1038/s41429-026-00924-0)
Supplement: Supplementary file 1 — SUPPLEMENTAL MATERIAL [file 41429_2026_924_MOESM1_ESM.pdf]

SUPPLEMENTAL MATERIAL

Discovery of Actinomycin Derivatives with Improved Selectivity against Malaria Parasites from a *Streptomyces* Culture Library

Aiko Teshima[1,2,†], Awet Alem Teklemichael[3,†], Asahi Hirata[1,2], Momoko Akimoto[1,2], Mayumi Taniguchi[3,4], Rukman Muslimin[1,5], Sho Ogaki[1,2], Alimuddin Ali[5], Toshihiro Suzuki[6], Shusaku Mizukami[3,4,*], and Kenji Arakawa[1,2,*]

[1] Program of Biotechnology, Graduate School of Integrated Sciences for Life, Hiroshima University, 1-3-1 Kagamiyama, Higashi-Hiroshima, Hiroshima 739-8530, Japan

[2] Hiroshima Research Center for Healthy Aging (HiHA), Hiroshima University, 1-3-1 Kagamiyama, Higashi-Hiroshima, Hiroshima 739-8530, Japan

[3] Department of Immune Regulation, Shionogi Global Infectious Diseases Division, Institute of Tropical Medicine, Nagasaki University, 1-12-4 Sakamoto, Nagasaki 852-8523, Japan

[4] School of Tropical Medicine and Global Health, Nagasaki University, 1-12-4 Sakamoto, Nagasaki 852-8523, Japan

[5] Department of Biology, Universitas Negeri Makassar, Jl. Dg Tata Raya Parangtambung, Makassar, South Sulawesi 90223, Indonesia

[6] Department of Fermentation Sciences, Faculty of Applied Biosciences, Tokyo University of Agriculture, 1-1-1 Sakuragaoka, Setagaya, Tokyo, 156-8502, Japan

* Correspondence: Kenji Arakawa (E-mail; karakawa@hiroshima-u.ac.jp), Shusaku Mizukami (E-mail; mizukami@nagasaki-u.ac.jp),

† These authors have contributed equally to this work.

Contents

Experimental procedure for antimalarial activity and cytotoxicity assay, total DNA and NGS

Sample preparation -- 3

Table S1. The ^{1}H- and ^{13}C-NMR assignments of actinomycin X_2 (**1**) -------------------------------- 8

Table S2. The ^{1}H- and ^{13}C-NMR assignments of actinomycin $X_{0\beta}$ (**2**) -------------------------------- 9

Table S3. Deduced functions of actinomycin biosynthetic gene (*actm*) cluster in

Streptomyces antibioticus strain HUT6035 --- 10

Figure S1. ^{1}H-NMR spectrum of actinomycin X_2 (**1**) -- 11

Figure S2. ^{13}C-NMR spectrum of actinomycin X_2 (**1**) -- 11

Figure S3. ^{1}H-NMR spectrum of actinomycin $X_{0\beta}$ (**2**) -- 12

Figure S4. ^{13}C-NMR spectrum of actinomycin $X_{0\beta}$ (**2**) -- 12

Parasite culture

Blood stages of *P. falciparum* 3D7 cell lines were provided by Nagasaki University with support in part by NEKKEN Bio-Resource Center (NEKKEN BRC), Institute of Tropical Medicine, Nagasaki University as a part of National BioResource Project (NBRP), MEXT, Japan. Human erythrocytes used for parasite culture were obtained from the Japan Red Cross Society (Registration No. 28J0060). The 3D7 parasites were cultivated in O+ erythrocytes in 2% hematocrit in Roswell Park Memorial Institute (RPMI) 1640-based complete medium (CM) supplemented with 5% AB+ human serum (prepared from plasma), 0.25% AlbuMax I (Gibco, Waltham, MA), 12.5 μg/mL gentamycin, and 200 mM hypoxanthine at 37°C (Ref. 1).

Cell culture

Primary adult mouse brain (AMB) cells were isolated and established at the NEKKEN Bio-Resource Center, according to previously established methods (Ref. 2). Briefly, the primary cells, which were passaged several times to be adapted to in vitro conditions, were maintained in Minimum Essential Medium (MEM) (Wako Pure Chemicals Industrial Ltd, Osaka, Japan) supplemented with 10% fetal bovine serum (FBS), penicillin/streptomycin solution (100 units/mL penicillin G and 100 mg/mL streptomycin sulfate) (Wako Pure Chemicals Industrial Ltd) and incubated at 37°C and 5% CO_2. For the cytotoxicity assay, primary cells that had completed three passages were used.

Antimalarial growth inhibition assays

The antimalarial growth inhibition assay to determine 50% inhibition concentration (IC_{50}) of compounds was performed as described before, with a minor modification (Ref. 3). Briefly, The *P. falciparum* cultures (0.75% parasitemia and 2% hematocrit) were seeded on a 96-well black plate with the clear bottom (Thermo Fisher Scientific, Rochester, NY) and

exposed to compounds serially diluted threefold over a concentration range of 2.5 μg/mL to 0.13 ng/mL at the final concentration. As a result of the dose-response assay, IC_{50} value, the concentration of drug required to reduce parasites by 50% ($10(\log^{(A/B) \times (50 - C)/(D - C) + \log(B)})$ was obtained, where A represents the lowest concentration at which the percentage inhibition was greater than 50%, B is the highest concentration value at which the percentage inhibition was less than 50%, C is the percentage inhibition value of the sample at concentration B, and D is the percentage inhibition value of the sample at concentration A. The culture plates were kept at 37°C under mixed gas (5% O_2, 5% CO_2, and 90% N_2) for 48 hrs. Each in vitro experiment was performed in duplicate wells and repeated twice. The parasite growth inhibition was obtained by dividing the test samples' parasitemia by the negative controls' average.

SYBR Green – I (Lonza, Rockland, ME) assay technique was used to detect the parasite DNA. The RBCs were lysed after 48 hrs of incubation by adding 100 μL of lysis buffer (20 mM Tris, 10 mM EDTA, 0.01% saponin (wt/vol), and 0.1% Triton X-100 (vol/vol), pH 7.5) and 1× final concentration of SYBR Green - I into each well. The plates were incubated at room temperature for 1 hr with gentle agitation. The relative fluorescence units (RFU) per well were then determined at 485–515 nm (filter) for 0.1 sec per exposure using a multilabel plate reader (ARVO 1430; Perkin Elmer, Waltham, MA, USA).

Cytotoxicity assay

Cytotoxicity assay was performed to determine 50% cytotoxic concentration (CC_{50}) as previously described with minor modification (Ref. 4). Briefly, AMB cells (1×10^4 cells) were seeded in a 96-well plate (black plate with a clear bottom) and incubated at 37°C in a CO_2 incubator for 24 hrs. Cells were exposed to compounds serially diluted threefold over 5 μg/mL – 0.25n g/mL and their negative controls were added, and the cells were further incubated for 48 hrs. To evaluate the cell viability (%), 10 μL of Alamar Blue solution (10%,

Funakoshi Co., Tokyo, Japan) was added into each well and the cells were incubated for 2 hrs at 37°C. The fluorescence intensity of each well was measured at 590 nm for 0.1 sec per exposure using a multi-label plate reader. CC_{50} value, the concentration of drug required to reduce cell viability by 50% ($10^{(\log(A/B) \times (50 - C)/(D - C) + \log(B))}$), was determined for samples that showed less than 50% viability in the initial screening, where A represented the lowest concentration value at which the percentage viable cell showed greater than 50% , B was the highest concentration value at which the percentage viable cell showed less than 50%, C was the percentage viable cell value of the sample at a concentration B, and D was the percentage viable cell value of the sample at a concentration A. All assays were performed twice independently in duplicate wells. The IC_{50} and CC_{50} values were used as indicators of *in vitro* antimalarial activity and cytotoxicity, respectively. The selectivity index (SI) was obtained by dividing the CC_{50} value by the IC_{50} value.

DNA sequencing and assembly

Streptomyces total DNA was prepared according to previously modified protocol (Ref. 5). The mycelium from a 50 ml culture was suspended in 13 ml of TSE–sucrose buffer (30 mM Tris, 50 mM NaCl, 5 mM EDTA at pH 8.0, and 10.3% sucrose), and then the resultant was treated with a mixture containing 1 ml of 0.5 M EDTA (pH 8.0) and 4 ml of lysozyme (5 mg/ml in TSE–sucrose) at 37 °C for 30 min. The resulting mixture was further treated with 2 ml of Actinase E (5 mg/ml in TSE, Kaken Seiyaku, Tokyo) at 37 °C for 30 min, and then with 2 ml of 10% sodium dodecyl sulfate for an additional 30 min. After additional treatment with 2 ml of 5 M NaCl for 30 min at 37 °C, the mixture was left at 4°C over-night. The resulting mixture was centrifuged at 12,000 rpm for 20 min, and its supernatant was subjected to ethanol precipitation. The syrupy pellet was collected by a sterile glass rod, and rinsed with 70% aqueous ethanol. The pellet dissolved in TE (10 mM Tris, 1 mM EDTA, pH 8.0), and the mixture was incubated with RNase (100 µg/ml) at 37 °C for 1 h, and extracted with

phenol–chloroform. The resulting supernatant was further subjected with ethanol precipitation and collection as above mentioned.

The quantity and purity of DNA were assessed using a Qubit 2.0 fluorometer (Thermo Fisher Scientific, Waltham, MA, United States) with a Qubit dsDNA BR Assay Kit (Thermo Fisher Scientific). A genomic DNA library was prepared using the NEBNext Ultra II FS DNA Library Prep Kit for Illumina (New England Biolabs) according to the manufacturer's instructions. Draft genome sequencing was performed using a paired-end sequencing strategy (2 × 300 bp) on an Illumina NextSeq 1000 platform (Illumina, Inc., San Diego, CA, USA). Adapter sequences and low-quality regions in the Illumina reads were trimmed using FastQC (Ref. 6) with default parameters. *De novo* assembly of the raw genome sequencing data was performed using SPAdes 4.2.0 (Ref. 7). Genome assembly quality was assessed using QUAST 5.3.0 (Ref. 8) and BUSCO v. 5.4.4 (Ref. 9).

References

(1) Teklemichael AA, Teshima A, Hirata A, Akimoto M, Taniguchi M, Khodakaramian G, et al. Discovery of antimalarial drugs from secondary metabolites in actinomycetes culture library. Trop Med Health. 2024;52:47.

(2) Teklemichael AA, Mizukami S, Toume K, Mosaddeque F, Kamel MG, Kaneko O, et al. Anti-malarial activity of traditional Kampo medicine Coptis rhizome extract and its major active compounds. Malar J. 2020;19:204.

(3) Hashim Y, Toume K, Mizukami S, Ge YW, Taniguchi M, Teklemichael AA, et al. Phenylpropanoid conjugated iridoids with anti-malarial activity from the leaves of Morinda morindoides. J Nat Med. 2021;75:915-925.

(4) Mizuta S, Mosaddeque F, Tun MMN, Teklemichael AA, Taniguchi M, Hosokawa M, et al. Challenges based on antiplasmodial and antiviral activitiesof 7-chloro-4-aminoquinoline derivatives. ChemMedChem. 2023;18:e202200586.

(5) Suwa M, Sugino H, Mori E, Sasaoka A, Fujii S, Shinkawa H, et al. Identification of two polyketide synthase gene clusters on the linear plasmid pSLA2-L in Streptomyces rochei. Gene. 2000;246:123–31.

(6) Chen S, Zhou Y, Chen Y, Gu J. Fastp: an ultra-fast all-in-one FASTQ preprocessor. Bioinformatics. 2018;34:i884−i890.

(7) Prjibelski A, Antipov D, Meleshko D, Lapidus A, Korobeynikov A. Using SPAdes De Novo Assembler. Curr Protoc Bioinformatics. 2020;70:e102.

(8) Gurevich A, Saveliev V, Vyahhi N, Tesler G. QUAST: quality assessment tool for genome assemblies. Bioinformatics. 2013;29:1072-75.

(9) Simão FA, Waterhouse RM, Ioannidis P, Kriventseva EV, Zdobnov EM. BUSCO: assessing genome assembly and annotation completeness single-copy orthologs. Bioinformatics. 2015;31:3210−2.

Table S1. The ^{1}H and ^{13}C NMR assignments of actinomycin X_2 (**1**)

	Ring A			Ring B		
	Position	δ_C, mult.	δ_H, mult. (J in Hz)	Position	δ_C, mult.	δ_H (, J in Hz)
L-Thr	1	168.6, C	–	1	168.9, C	–
	2	55.0, CH	4.56, m	2	54.7, CH	4.48, m
	3	74.7, CH	5.16, m	3	74.6, CH	5.25, m
	4	17.1, CH_3	1.14, d (6.5)	4	17.7, CH_3	1.27, d (6.0)
	NH		7.17, d (6.5)	NH		7.71, d (5.5)
D-Val	1	173.5, C	–	1	174.0, C	–
	2	58.4, CH	3.58, dd (9.0, 5.5)	2	57.1, CH	3.73, dd (9.5, 5.5)
	3	31.8, CH	2.11, m	3	31.6, CH	2.09, m
	4	18.9, CH_3	0.91, d (7.0)	4	18.8, CH_3	0.89, d (8.0)
	5	19.2, CH_3	1.12, d (6.5)	5	19.0, CH_3	1.15, d (6.5)
	NH	–	7.66, d (6.0)	NH	–	8.19, d (6.0)
L-Pro (ring A)	1	173.1, C	–	1	172.7, C	–
4-Oxo-L-Pro (ring B)	2	56.3, CH	5.95, d (9.0)	2	54.2, CH	6.58, d (10.5)
	3	31.0, CH_2	2.78, m; 1,86, m	3	41.9, CH_2	3.89, m; 2.35, m
	4	22.9, CH	2.24, m	4	208.8, C	–
	5	47.4, CH_2	3.91, m; 3.73, m	5	52.8, CH_2	4.57, m; 3.91, m
Sar	1	166.0, C	–	1	165.9, C	–
	2	51.3, CH_2	4.72, d (17.5); 3.66, d (17.0)	2	51.3, CH_2	4.60, d (16.5); 3.66, d (17.0)
	NMe	34.7, CH_3	2.89, s	NMe	34.9, CH_3	2.90, s
N-Me-L-Val	1	166.4, C	–	1	166.3, C	–
	2	71.2, CH	2.69, m	2	71.4, CH	2.69, m
	3	26.9, CH	2.69, m	3	26.9, CH	2.69, m
	4	21.5, CH_3	0.96, d (5.5)	4	21.7, CH_3	0.99, d (5.5)
	5	19.0, CH_3	0.75, d (5.5)	5	19.0, CH_3	0.76, d (5.0)
	NMe	31.6, CH_3	2.93, s	NMe	31.8, CH_3	2.94, s
Phenoxazinone	1	101.6, C	–			
	2	147.5, C	–			
	3	179.0, C	–			
	4	113.6, C	–			
	4a	145.0, C	–			
	5a	140.4, C	–			
	6	127.8, C	–			
	7	130.3, CH	7.38, d (7.5)			
	8	126.1, CH	7.62, d (8.0)			
	9	132.0, C	–			
	9a	129.1, C	–			
	10a	145.9, C	–			
	11	167.5, C	–			
	12	7.7, CH_3	2.20, s			
	13	15.0, CH_3	2.56, s			
	14	172.7, C	–			

Table S2. The ^{1}H and ^{13}C NMR assignments of actinomycin $X_{0\beta}$ (**2**)

	Ring A			Ring B		
	Pos.	δ_C, mult.	δ_H, mult. (J in Hz)	Pos.	δ_C, mult.	δ_H, mult. (J in Hz)
L-Thr	1	168.6, C	–	1	168.8, C	–
	2	55.4, CH	4.56, dd (6.6, 2.4)	2	54.8, CH	4.48, dd (6.6, 3.0)
	3	74.6, CH	5.25, m	3	75.2, CH	5.25, m
	4	17.7, CH_3	1.29, d (6.0)	4	17.6, CH_3	1.25, d (5.4)
	NH		7.44, d (6.0)	NH		7.49, d (7.2)
D-Val	1	173.4, C	–	1	173.1, C	–
	2	58.7, CH	3.56, m	2	58.0, CH	3.56, m
	3	31.9, CH	2.13, m	3	31.6, CH	2.10, m
	4	19.3, CH_3	0.91, d (6.6)	4	19.2, CH_3	0.89, d (6.6)
	5	19.1, CH_3	1.12, d (6.6)	5	18.9, CH_3	1.14, d (6.6)
	NH		8.17, d (5.4)	NH		7.90, d (6.6)
L-Pro (ring A) 4-Hydroxy-L-Pro (ring B)	1	173.0, C	–	1	172.9, C	–
	2	56.3, CH	6.00, d (9.0)	2	56.9, CH	6.08, dd (9.0, 2.4)
	3	31.0, CH_2	2.79, m; 1,85, dd (11, 6.6)	3	54.7, CH_2	4.15, dd (13, 4.8); 3.98, dd (13, 7.8)
	4	22.9, CH	2.06, m	4	70.0, CH	4.70, m
	5	47.5, CH_2	3.86, m; 3.74, m	5	54.7, CH_2	4.15, dd (5.4, 13); 3.98, dd (7.2, 13)
Sar	1	166.3, C	–	1	166.1, C	–
	2	51.3, CH_2	4.74, d (17.4); 3.65, d (17.4)	2	51.2, CH_2	4.55, d (17.4); 3.61, d (17.4)
	NMe	35.0, CH_3	2.89, s	NMe	35.0, CH_3	2.89, s
N-Me-L-Val	1	167.5, C	–	1	166.4, C	–
	2	71.3, CH	2.70, m	2	71.4, CH	2.72, d (9.0)
	3	26.9, CH	2.69, m	3	27.0, CH	2.69, m
	4	21.6, CH_3	0.98, d (6.0)	4	21.7, CH_3	0.96, d (6.0)
	5	19.0, CH_3	0.76, d (6.6)	5	19.0, CH_3	0.75, d (6.6)
	NMe	39.5, CH_3	2.96, s	NMe	39.2, CH_3	2.95, s
Phenoxazinone	1	102.6, C	–			
	2	147.0, C	–			
	3	179.0, C	–			
	4	113.5, C	–			
	4a	145.0, C	–			
	5a	140.6, C	–			
	6	128.2, C	–			
	7	129.0, CH	7.37, d, 7.2)			
	8	126.2, CH	7.67, d, 7.8)			
	9	131.3, C	–			
	9a	129.5, C	–			
	10a	146.0, C	–			
	11	167.5, C	–			
	12	7.8, CH_3	2.25 (s)			
	13	15.1, CH_3	2.55 (s)			
	14	172.9, C	–			

Table S3. Deduced functions of actinomycin biosynthetic gene (*actm*) cluster in *Streptomyces antibioticus* strain HUT6035

Gene products in *actm* cluster in HUT6035	Number of amino acid	Predicted function	Gene products in an *saacm* cluster in *S. antibioticus* IMRU3720	Number of amino acid for homologous gene products in the *saacm* cluster	ID/SI to the *saacm* gene products (%) *
orf (-3)	223	LysE family translocator	*orf* (-3)	223	97/97
orf (-2)	148	ankyrin repeat domain-containing protein	*orf* (-2)	148	98/98
orf (-1)	2301	glycoside hydrolase family 92 protein	*orf* (-1)	2288	98/98
actmT	211	DUF6875 domain-containing protein	*saacmT*	211	99/99
actmS	187	hypothetical protein	*saacmS*	187	99/99
actmR	66	MbtH family protein	*saacmR*	66	98/100
actmD	78	hypothetical protein	*saacmD*	68	99/100
actmA	467	FadD3 family acyl-CoA ligase	*saacmA*	467	98/98
actmB	2611	non-ribosomal peptide synthetase	*saacmB*	2611	99/99
actmC	4235	non-ribosomal peptide synthetase	*saacmC*	4235	98/98
actmE	210	DUF6875 domain-containing protein	*saacmE*	210	99/99
actmF	317	alpha/beta hydrolase	*saacmF*	310	95/96
actmG	287	tryptophan 2,3-dioxygenase	*saacmG*	287	99/99
actmH	420	kynureninase	*saacmH*	420	99/99
actmI	346	methyltransferase	*saacmI*	346	99/99
actmM	433	cytochrome P450	*saacmM*	433	99/99
actmN	68	ferredoxin	*saacmN*	68	100/100
actmJ	215	LmbU family transcriptional regulator	*saacmJ*	219	100/100
actmU	278	TetR/AcrR family transcriptional regulator C-terminal domain-containing protein	*saacmU*	280	99/99
actmV	294	siderophore-interacting protein	*saacmV*	294	99/99
actmW	327	ATP-binding cassette domain-containing protein	*saacmW*	327	99/99
actmX	255	ABC transporter permease	*saacmX*	255	99/100
actmY	753	excinuclease ABC subunit UvrA	*saacmY*	753	99/99
orf (+1)	699	WD40 repeat domain-containing serine/threonine protein kinase	*orf* (+1)	699	98/98
orf (+2)	368	alpha/beta hydrolase	*orf* (+2)	330	98/99
orf (+3)	216	NAD(P)H-dependent oxidoreductase	*orf* (+3)	79	92/94

* ID/SI: Identity/similarity

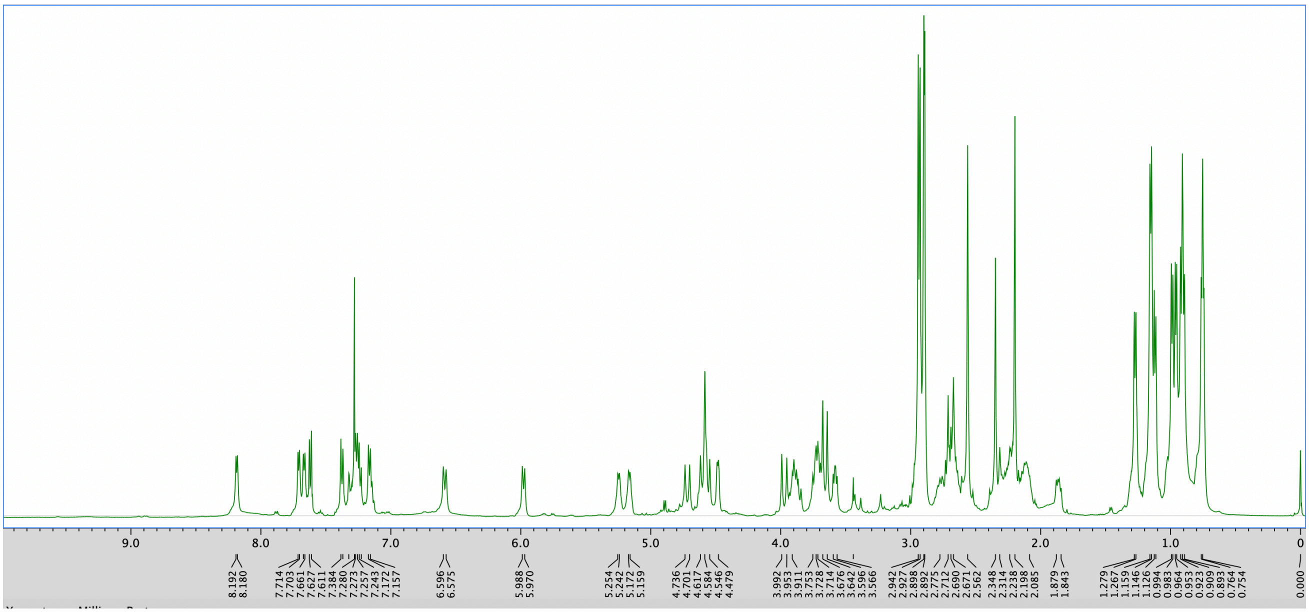

Figure S1. ^{1}H-NMR spectrum of actinomycin X_2 (**1**)

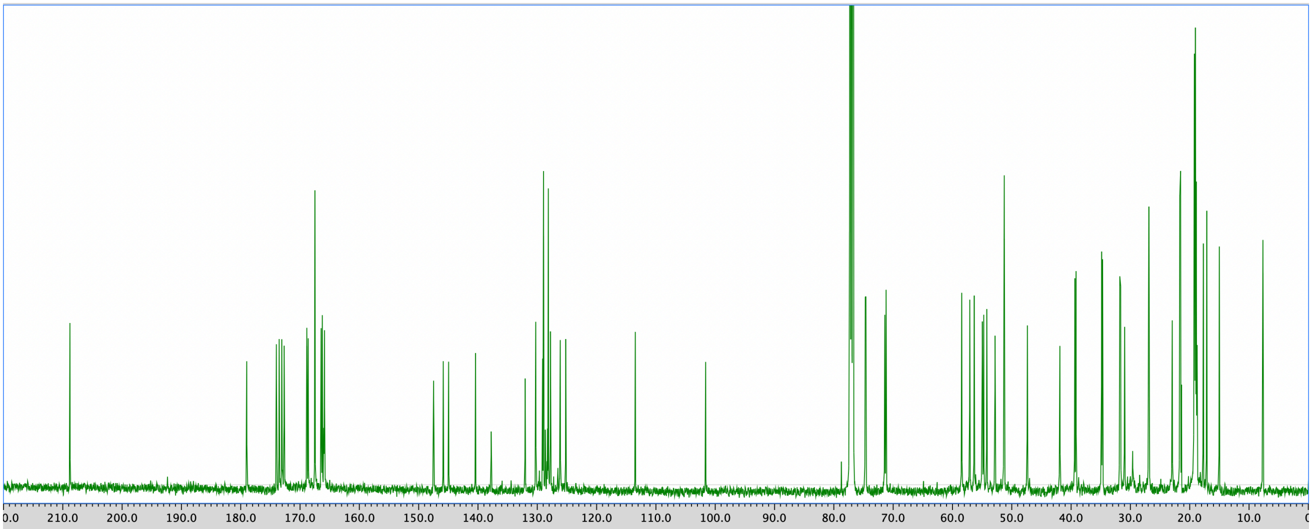

Figure S2. ^{13}C-NMR spectrum of actinomycin X_2 (**1**)

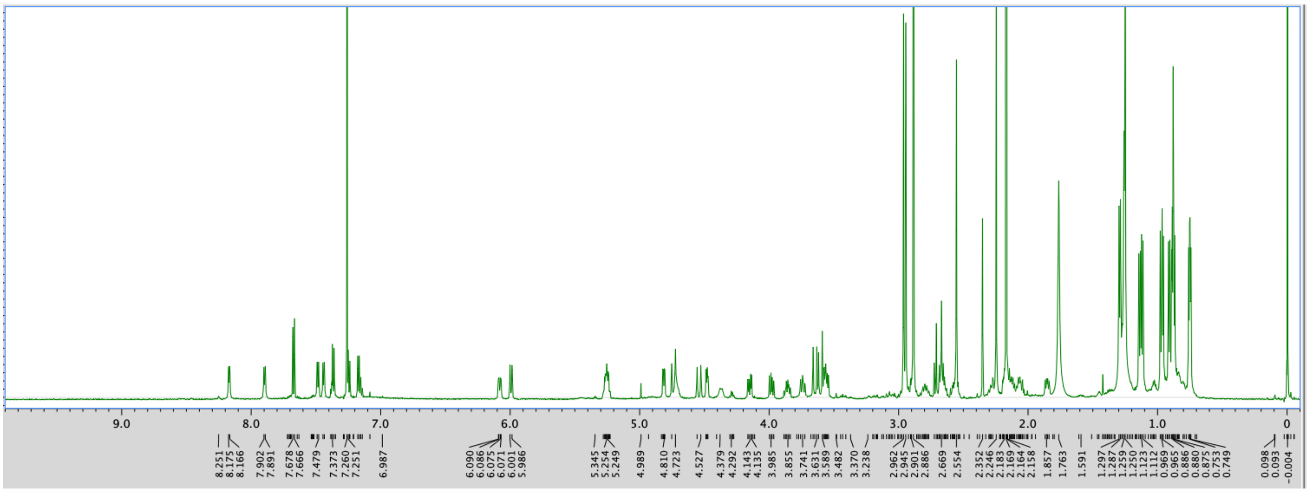

Figure S3. ^{1}H-NMR spectrum of actinomycin $X_{0\beta}$ (**2**)

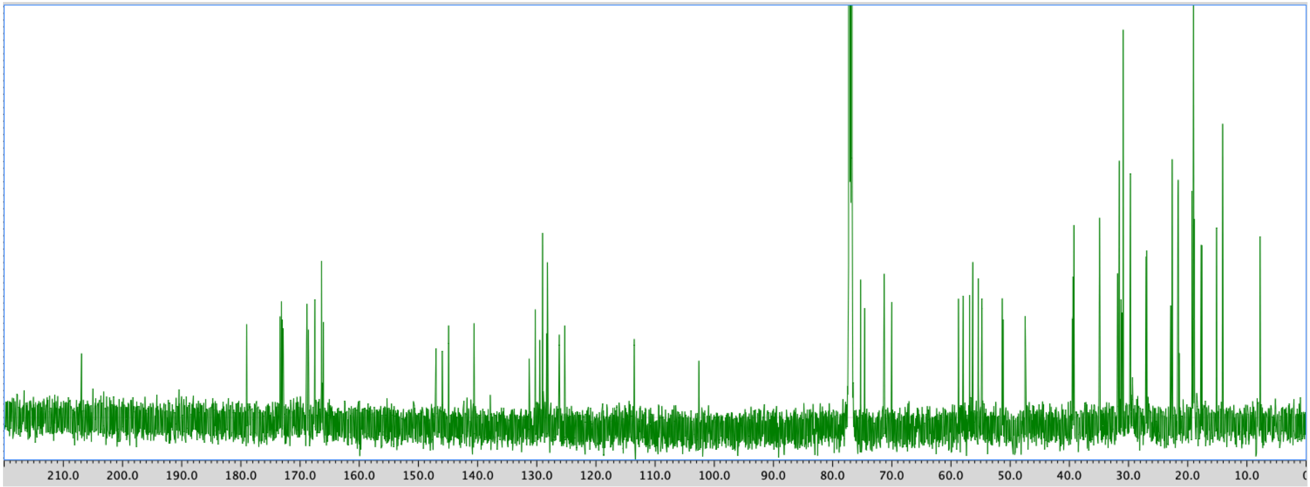

Figure S4. ^{13}C-NMR spectrum of actinomycin $X_{0\beta}$ (**2**)
